# Severe Multi-Organ Failure and Hypereosinophilia: When to Call It “Idiopathic”?

**DOI:** 10.1177/2324709618758347

**Published:** 2018-02-15

**Authors:** Massimo Radin, Luca Bertero, Dario Roccatello, Savino Sciascia

**Affiliations:** 1 Center of Research of Immunopathology and Rare Diseases, Coordinating Center of Piemonte and Valle d’Aosta Network for Rare Diseases, Department of Clinical and Biological Sciences, and SCDU Nephrology and Dialysis, University of Turin and S. Giovanni Bosco Hospital, Turin, Italy; 2 Pathology Division, AOU Cittàdella Salute e della Scienza di Torino, Turin, Italy

**Keywords:** IHES, idiopathic hypereosinophilic syndrome, hypereosinophilia

## Abstract

The hypereosinophilic syndrome is a rare disease characterized by the association between high absolute eosinophil count and eosinophil-mediated organ damage. We describe a case of a 70-year-old male with an absolute eosinophil count of 2130 cells/µL. Clinical symptoms and signs included the following: severe asthenia, axonal sensitive motor neuropathy, basal pleural effusion with signs of hypoventilation on chest radiography, and gastrointestinal symptoms such as severe diarrhea, weight loss (−10 kg in 6 months), abdominal pain, and vomiting. On physical examination he had an urticarial dermatitis on his back, abdomen, and lower limbs. An extensive instrumental and laboratory diagnostic workup was performed. When all causes of primary and secondary hypereosinophilic syndrome were excluded, treatment with solumedrol infusion and oral prednisone was started, with a rapid recover of clinical symptoms and normalization of laboratory parameters. A complete remission of the laboratory and clinical findings was achieved after 2 months and maintained over 1-year follow-up.

## Background

Hypereosinophilia (HE) is defined in the peripheral blood as an absolute eosinophil count >1500 cells/µL, confirmed on 2 examinations and/or pathological confirmation of HE on tissue.^1^ The hypereosinophilic syndrome (HES) is a rare disease characterized by the association between HE and eosinophil-mediated organ infiltration and damage or dysfunction. Clinical presentation of patients might be very heterogeneous since it is strictly correlated to organ damage mediated by eosinophils. Symptoms can be insidious, and HES might be overlooked; however, in some patients the evolution of cardiovascular or neurological complication might be swift and life-threatening.

In idiopathic HES (IHES), the underlying cause of HE remains unknown despite investigations and complete etiological workup.^2^ When all causes of primary and secondary HES are excluded, treatment is generally warranted.^3^

## Case Presentation

**Figure 1.**
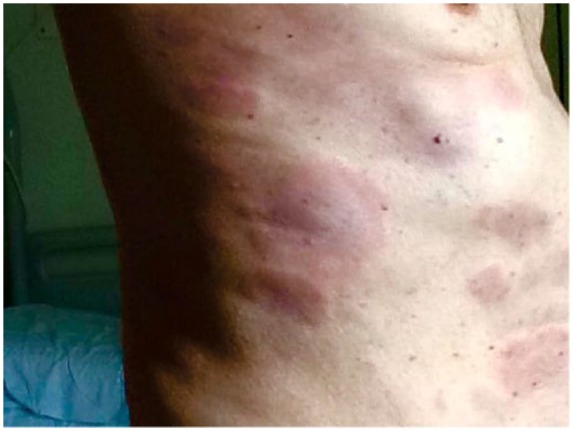
Urticarial dermatitis on back and abdomen, left side.

A 70-year-old male, with history of rheumatic pericarditis in childhood and no family history of lymphoproliferative and autoimmune diseases, was taken to the emergency department for neuralgic pain in both feet and in the lumbosacral region. Electromyography (EMG) showed axonal sensitive motor neuropathy. He also complained of a persistent nonproductive cough over the previous month before the admission. On physical examination he had an urticarial dermatitis on his back, abdomen, and lower limbs, more marked on the left side (Figure 1). After a preliminary workout, he was referred to our center. When he came to our attention, after 6 months since the onset of the first clinical manifestation, the sensitive motor neuropathy had worsened, especially in the left leg, compromising the deambulation of the patient. Furthermore, he reported severe asthenia and a further deterioration in his gastrointestinal symptoms including severe diarrhea, weight loss (over 10 kg), abdominal pain, and vomiting. On physical examination the urticarial dermatitis had spontaneously resolved. At admission in our center, he was not receiving any treatment.

## Investigations

When the patient came to our attention the initial blood count highlighted an absolute eosinophil count of 2130 cells/µL with 7900 white blood cells/µL (relative eosinophil count 27%) with normal differential. Liver and renal function tests, vitamin B_12_ level, complement, prothrombin time, serum protein electrophoresis, angiotensin-converting enzyme, and serum tryptase were all within normal range.

**Figure 2.**
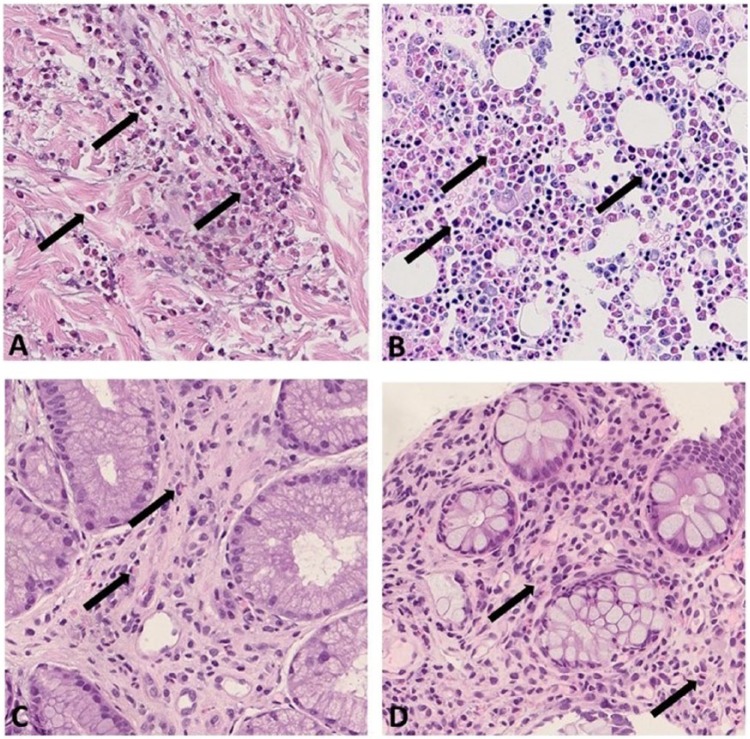
Histological images showing significant eosinophilic infiltrates (arrows) at multiple sites: perivascular and interstitial in skin derma (A: hematoxylin and eosin (H&E) 40×), bone marrow (B: Dominici 40×), gastric antrum (C: H&E 40×), and sigmoid colon (D: H&E 40×).

Indirect parameters of inflammations were elevated: erythrocyte sedimentation rate of 87 mm, C-reactive protein of 2 mg/dL, and lactate dehydrogenase of 289 UI/L. Autoimmunity (antinuclear antibodies [ANA] screening, anti-neutrophil cytoplasmic antibody, and cryoglobulins) and JAK2 V617F mutation were negative. EMG showed a considerable deterioration of the axonal sensitive motor neuropathy compared with the EMG taken 6 months earlier. Table 1 summarizes the performed investigations, and Figure 2 shows the histological images showing significant multi-organ eosinophilic infiltrates.

**Table 1.** Previous Investigations Undergone by the Patient.

Blood count	WBC 1003 (cells/µL), neutrophils 5040, lymphocytes 1230, monocytes 25, eosinophils 3490, basophiles 10, RBC 4500, Hb 13.5 g/dL, MCV 93 fL, and PLTs 217 000
Blood tests	Immunoglobulins (Ig): IgG 1080/IgM 71/IgA 187, C3 68 mg/dL, C4 7 mg/dL, and ACE 61.2 U/L
	ANA negative, anti-dsDNA negative, ANCA negative, cryoglobulins negative, antiphospholipid antibodies negative, and rheumatoid factor 472
	HBV-DNA: negative; HCV-RNA: negative
	Normal complete urine test
	Parvovirus IgG/IgM: negative; anti-*Borrelia burgdoferi*: negative
	Serum tryptase level: within normal range
	Blood smear: no circulating blasts, dysplastic cells, and monocytosis
Lymphocyte phenotyping and molecular analysis	Molecular studies including screening for FIP1L1-PDGFRA: negative
	Peripheral blood lymphocyte phenotyping and T-cell receptor gene rearrangement studies: absence of phenotypically abnormal and/or clonal T lymphocytes
Parasitology	Serology negative for *Strongyloides stercoralis, Schistosoma* spp, *Toxocara* species, and filaria Stool examination: negative for multiple stool ova and parasite testing (eg, hookworm, *Schistosoma* species)
Skin biopsy	Perivascular and both superficial and deep interstitial urticarial dermatitis with eosinophilic prevalence
	No sign of leucocytoclastic vasculitis
	IF: negative
Bone marrow biopsy and cytogenetic	CD34+ cells: 0.4%
	No immunophenotypical alterations of blast cells
	Expansion cytogenetic: absence of 5q33, 4q12, or 8p11.2 translocations
Lumbar puncture	Total proteins 43 mg/dL, glucose 48 mg/dL, 1 cellular element, viral DNA and RNA negative, cultural tests negative
	Isoelectrofocusing unremarkable
Vertebral column MRI	Hypointense T1 signaling of bone marrow all across the sections analyzed, possible expression of bone marrow hypercellularity
Abdominal echography	Thickened sigmoid colon’s walls (dimension between 6 and 8 mm)
	Prostate hypertrophia
Chest radiography	Basal pleural effusion with signs of hypoventilation of the surrounding parenchyma
Echocardiography	Low-grade mitral insufficiency
GI endoscopy and gastric biopsy	Endoscopy unremarkable
	Gastric biopsy: antral chronic gastritis with focused implement of eosinophils
24-hour blood pressure monitoring	Unremarkable
Total body CT scan	Bilateral basal pleural effusion (dimensions maximum: 20 mm) associated with areas of parenchyma’s hypoventilation
	Multiple lymph nodes in the mediastinum area (maximum diameter dimensions: 12, 17, and 26 mm)
	Diverticulosis of the sigmoid colon portion with no evident signs of alterations or ongoing inflammation
	Prostate hypertrophia
Colonoscopy and polyp biopsy	Diverticulosis of the sigmoid colon and sessile polyp (dimension 4 mm)
	Biopsy of the sessile polyp: colic mucous membrane with increased quota of eosinophils, tubular adenoma with low-grade dysplasia
Total body PET	No evidence of highly active metabolic disease

Abbreviations: WBC, white blood cell; RBC, red blood cell; Hb, hemoglobin; MCV, mean corpuscular volume; PLT, platelet; ANA, antinuclear antibodies; anti-dsDNA, anti-double stranded DNA; ANCA, anti-neutrophil cytoplasmic antibody; HBV-DNA, hepatitis B virus DNA; HCV-RNA; hepatitis C virus RNA; FIP1L1-PDGFRA, fip1-like1-platelet-derived growth factor receptor alpha gene; IF, immunofluorescence; CD, cluster of differentiation; MRI, magnetic resonance imaging; GI, gastrointestinal; CT, computed tomography; PET, positron emission tomography.

Since both secondary and clonal eosinophilia have been ruled out as possible diagnoses, a probable diagnosis of IHES was made.

## Differential Diagnosis

Categories of HES are subclassified according to the pathogenic mechanisms resulting in eosinophil expansion: primary, secondary, or idiopathic (when the underlying cause of HE remains unknown).

In primary HES, the eosinophilic expansion is due to an underlying clonal stem cell neoplasm (myeloid or eosinophilic). On the other hand, in the case of secondary HES, the eosinophilic expansion is driven by overproduction of eosinophilopoietic cytokines by other cell types and is polyclonal. This is the case in parasitic infections, certain solid tumors, and T-cell lymphoma, and the HE, when severe, can cause organ damage and dysfunction.

Furthermore, one should bear in mind that there are specific syndromes associated with HE, in which the role of eosinophils to the clinical presentation of the disease is still unknown, such as eosinophilic granulomatosis and polyangiitis and certain immunodeficiencies.

Table 2 summarizes the clinical and laboratory features of HES variants.

**Table 2.** Clinical and Laboratory Features of HES Variants.

HES Variants	Clinical and Laboratory Features	Possible Subtypes
Myeloproliferative variants	↑ Serum B_12_	PDGFRB and FGFR1 rearrangements
	Anemia and/or thrombocytopenia	JAK2 point mutation and translocation
	Hepatomegaly and/or splenomegaly	Chronic eosinophilic leukemia
	Circulating leukocyte precursors	PDGFRA or PDGFRB rearrangements
T-cell lymphocytic variants (LHES)	Prominent skin involvement	Aberrant IL5 producing T cells
	Polyclonal hypergammaglobulinemia	
	A progression to T-cell lymphoma might occur	
Familial HES	Asymptomatic eosinophilia	Autosomal dominant, mapped to 5q 3133
Idiopathic HES	Heterogeneous organ damage	
Organ restricted HES	Peripheral blood eosinophilia associated with single organ involvement	
Syndromes associated with hypereosinophilia	Underlying disorder associated with eosinophilia	Episodic angioedema with eosinophilia
		Eosinophilic granulomatosis with polyangiitis
		Other disorders associated with immune dysregulation

Abbreviations: HES, hypereosinophilic syndrome; LHES, lymphocytic variant HES; PDGFRB, platelet-derived growth factor receptor beta; FGFR1, fibroblast growth factor receptor 1; JAK2, Janus kinase 2; PDGFRA, platelet-derived growth factor receptor alpha.

## Treatment

**Figure 3.**
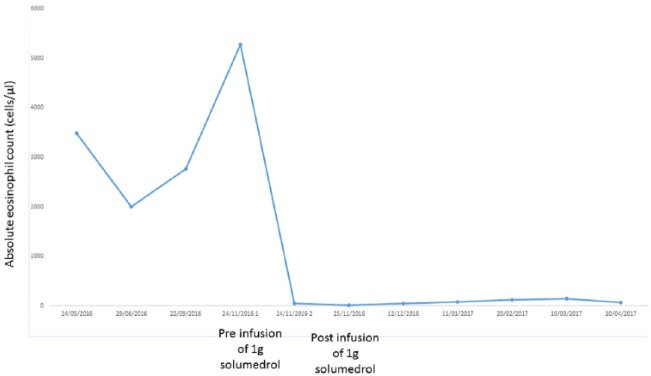
Absolute eosinophils count during time.

The patient was treated with 3 infusions of 1 g of methylprednisolone in 3 consecutive days. Oral prednisone was introduced with a dose of 50 mg, followed by a slow tapering with a maintenance dose of 10 mg for 8 weeks. The patient showed complete remission of the laboratory parameters after the first infusion of methylprednisolone, with an absolute eosinophil count of 50 cells/µL with 9240 white blood cells/µL (relative eosinophil count = 0.005%; Figure 3).

## Outcome and Follow-up

Oral prednisone was slowly tapered down to 5 mg in a 2-month period. The patient was closely monitored with weekly blood counts.

The absolute eosinophil count remained under 140 cells/µL over the year of follow-up. After the 2 infusions of methylprednisolone the patient had a prompt resolution of the asthenia and nausea. After 1 month, the chest radiography showed a resolution of the basal pleural effusion and showed no signs of hypoventilation of the surrounding parenchyma. The patient gained the weight that he had lost in the past, with resolution of diarrhea and abdominal pain.

The deambulation of the patient was improved; however, episodes of neuralgia in the left leg persisted. After the 2-month period, the oral prednisone was tapered down to 5 mg and deambulation of the patient was improved. After 4 months, EMG showed a net improvement of the axonal sensitive motor neuropathy with a resolution of the clinical symptoms.

## Discussion

IHES is a rare disorder, characterized by sustained HE, where the underlying cause of HE is unclear despite thorough etiologic investigations. When organ damage, mediated by eosinophilic infiltration and mediators, is associated to IHES, therapeutic intervention is warranted.

Prospective studies investigating IHES are still lacking, and to date, only single retrospective studies on a natural history of HE have been performed.^4,5^ Furthermore, the vast majority of reported patients with HE in these retrospective studies had well-defined causes of HE after appropriate etiologic workup, and only a small minority of cases was actually idiopathic. An appropriate diagnostic workup is crucial for a tailored management, as patients with IHES benefit from steroids, as shown in a retrospective cohort study by Ang et al.^4^

Recent research in cellular and molecular biology is leading to further characterization of distinct underlying hematological disorders in some patients with IHES. In fact, there have been a small number of reports documenting clonal populations of mature eosinophils in patients with IHES,^6,7^ but there they represent a limited minority of cases in the vast spectrum of this disease.

There is still an unmet need for future prospective studies involving this patient population, especially with regard to long-term follow-up and further clinical and laboratory characterizations. The lack of studies is the main reason of no clear consensus regarding therapy introduction and gold standard therapeutic intervention for these patients. The risk of IHES relapse after initial treatment in a long follow-up observation also remains unknown. Similarly, while the use of other immusuppressants could be considered as steroid sparing agents, their use in this setting still needs further investigation. Besides, in our case the lack of new clinical or laboratory sign of relapse after oral tapering down to 5 mg in a 2-month period did not support in our opinion the use of any further therapy.

Our patient is still laboratory and clinically monitored on a monthly basis, with an oral dose of prednisone tapered down to 5 mg. All organ involvement had completely resolved, with the exception of the sensitive motor neuropathy, which at 1-year follow-up, is currently in remission.
